# Supplementary material for: Short-term molecular consequences of chromosome mis-segregation for genome stability
Source: Nat Commun. 2023 Mar 11;14:1353. doi: 10.1038/s41467-023-37095-7 (PMC10008630; doi:10.1038/s41467-023-37095-7)
Supplement: Supplementary file 1 — Supplementary Information [file 41467_2023_37095_MOESM1_ESM.pdf]

# SUPPLEMENTARY INFORMATION FOR:
# Short-term molecular consequences of chromosome mis-segregation for genome stability

Lorenza Garribba[1, #], Giuseppina De Feudis[1, #], Valentino Martis[1], Martina Galli[2], Marie Dumont[3], Yonatan Eliezer[4], René Wardenaar[5], Marica Rosaria Ippolito[1], Divya R Iyer[6], Andréa E Tijhuis[5], Diana CJ Spierings[5], Michael Schubert[5], Silvia Taglietti[1], Chiara Soriani[1], Simon Gemble[3], Renata Basto[3], Nick Rhind[6], Floris Foijer[5], Uri Ben-David[4], Daniele Fachinetti[3], Ylli Doksani[2] and Stefano Santaguida[1,7,8,*]

1. Department of Experimental Oncology at IEO, European Institute of Oncology IRCCS, Via Adamello 16, 20139 Milan, Italy
2. IFOM ETS - The AIRC Institute of Molecular Oncology, via Adamello 16, 20139, Milan, Italy
3. Institut Curie, PSL Research University, CNRS, UMR144, Paris, France
4. Department of Human Molecular Genetics and Biochemistry, Faculty of Medicine, Tel Aviv University, Tel Aviv, Israel
5. European Research Institute for the Biology of Ageing, University of Groningen, University Medical Center Groningen, 9713 AV, Groningen, the Netherlands
6. Department of Biochemistry and Molecular Biotechnology, University of Massachusetts Chan Medical School, 364 Plantation Street, Worcester, MA 01605, USA
7. Department of Oncology and Hemato-Oncology, University of Milan, Via Santa Sofia 9/1, 20122 Milan, Italy
8. Lead Contact
#These authors contributed equally
*Correspondence: stefano.santaguida@ieo.it (SS)

**The PDF includes:**

**Supplementary figures:**

- Supplementary Fig.1: Impact of thymidine block and reversine treatment on DNA damage accumulation.
- Supplementary Fig. 2: Impact of mimosine treatment and Mps1 inhibition on cell cycle and DNA damage.

- Supplementary Fig.3: DDK inhibition leads to exacerbation of replication stress particularly in aneuploid cells.
- Supplementary Fig.4: Effect of RO3306 treatment on cell cycle and DNA damage.
- Supplementary Fig.5: POLD3 depletion and consequent MiDAS inhibition result in a further increase in aneuploid cell genome instability.
- Supplementary Fig.6: RNF168 asymmetric partitioning between daughter cells occurs at higher frequency in cells with reduced proliferative capacity.
- Supplementary Fig.7: Separation and characterization of aneuploid cycling and arrested cells.
- Supplementary Fig.8: Kinetics of DNA damage repair in aneuploid cells and respective controls upon IR exposure.

**Supplementary Table:**

- Supplementary Table 1: List of genes differentially expressed in arrested vs. cycling aneuploid cells.

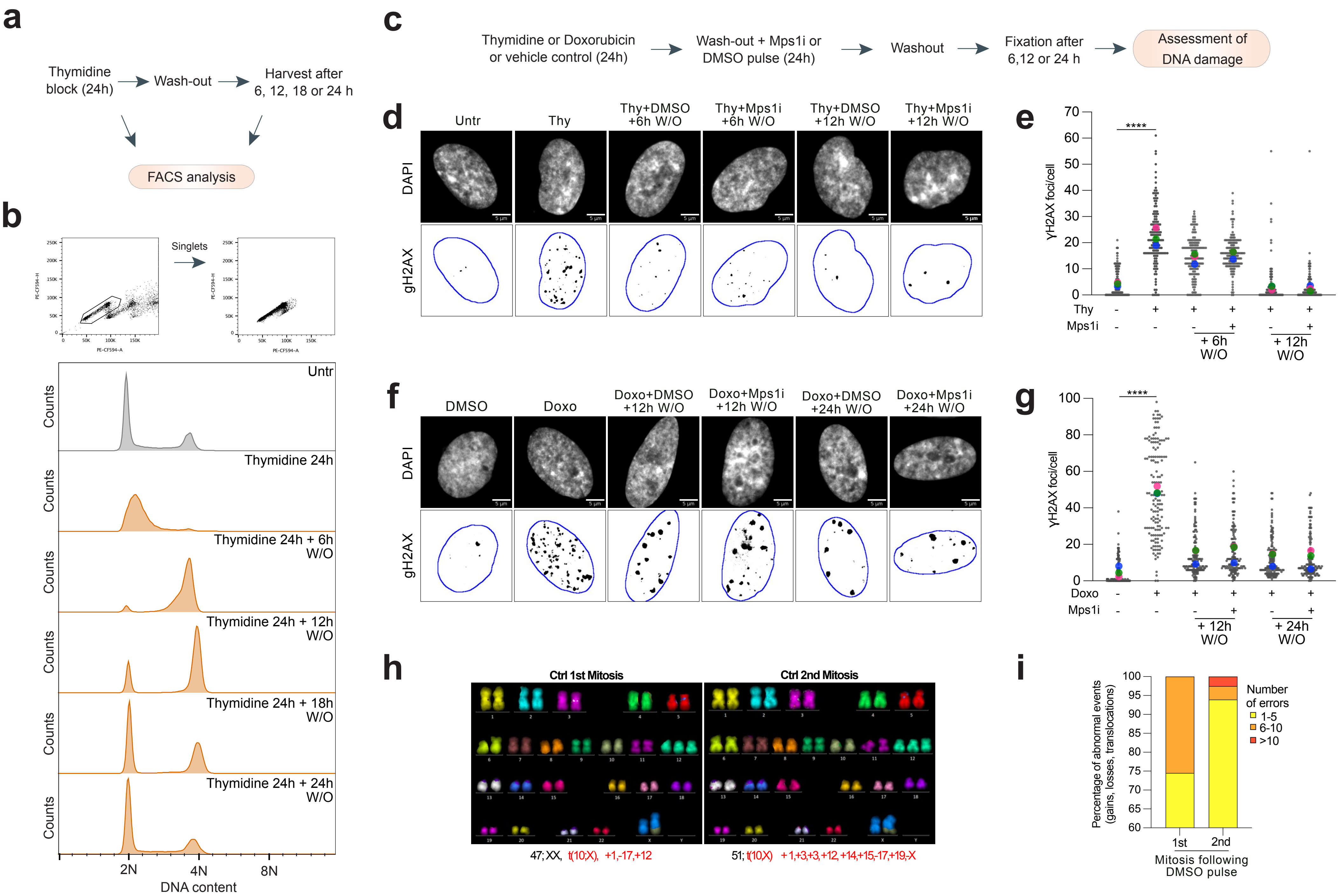


Supplementary Figure 1

**Supplementary Fig. 1: Impact of thymidine block and reversine treatment on DNA damage accumulation.** **a**, Experimental setup for the FACS analysis of thymidine treated cells. **b**, Representative flow cytometry profiles of propidium-iodide stained cells. Gating strategy is illustrated on top. **c**, Experimental setup for the analysis of DNA damage upon thymidine block or doxorubicin treatment. **d,e**, Representative images (d) and relative quantification (e) of γH2AX foci per cell (n=150 for each sample). **** indicates p<0,0001. **f,g**, Representative images (f) and relative quantification (g) of γH2AX foci per cell (n=150 for each sample). **** indicates p<0,0001. **h,i,** Representative mFISH images of karyotypes obtained from the $1^{st}$ (n=77) and the $2^{nd}$ mitosis (n=101) in control cells (h) and relative quantification of percentage of cells with chromosome gains, losses or translocations (i). Y axis shown from 60% to 100% for clarity (from 0 to 60% - and above - cells have 1-5 abnormal events, as indicated in key legend). T(10,X) and +12 were excluded from the analysis as they are clonal in hTERT RPE-1 cells. Untr, untreated, Thy, thymidine. Mps1i, Mps1 inhibitor. W/O, washout. Doxo, doxorubicin. Ctrl, control (DMSO pulsed). Scale bars, 5µm. LUT was inverted for γH2AX images. Blue borders in images are based on DAPI staining and define nuclei. Data are means of three biological replicates. Two-tailed unpaired Student's t-test was performed for data in panels e and g. In graphs, average values for each biological replicate are shown by colored dots (each color corresponds to a different biological replicate). Source Data are provided as a Source Data file.

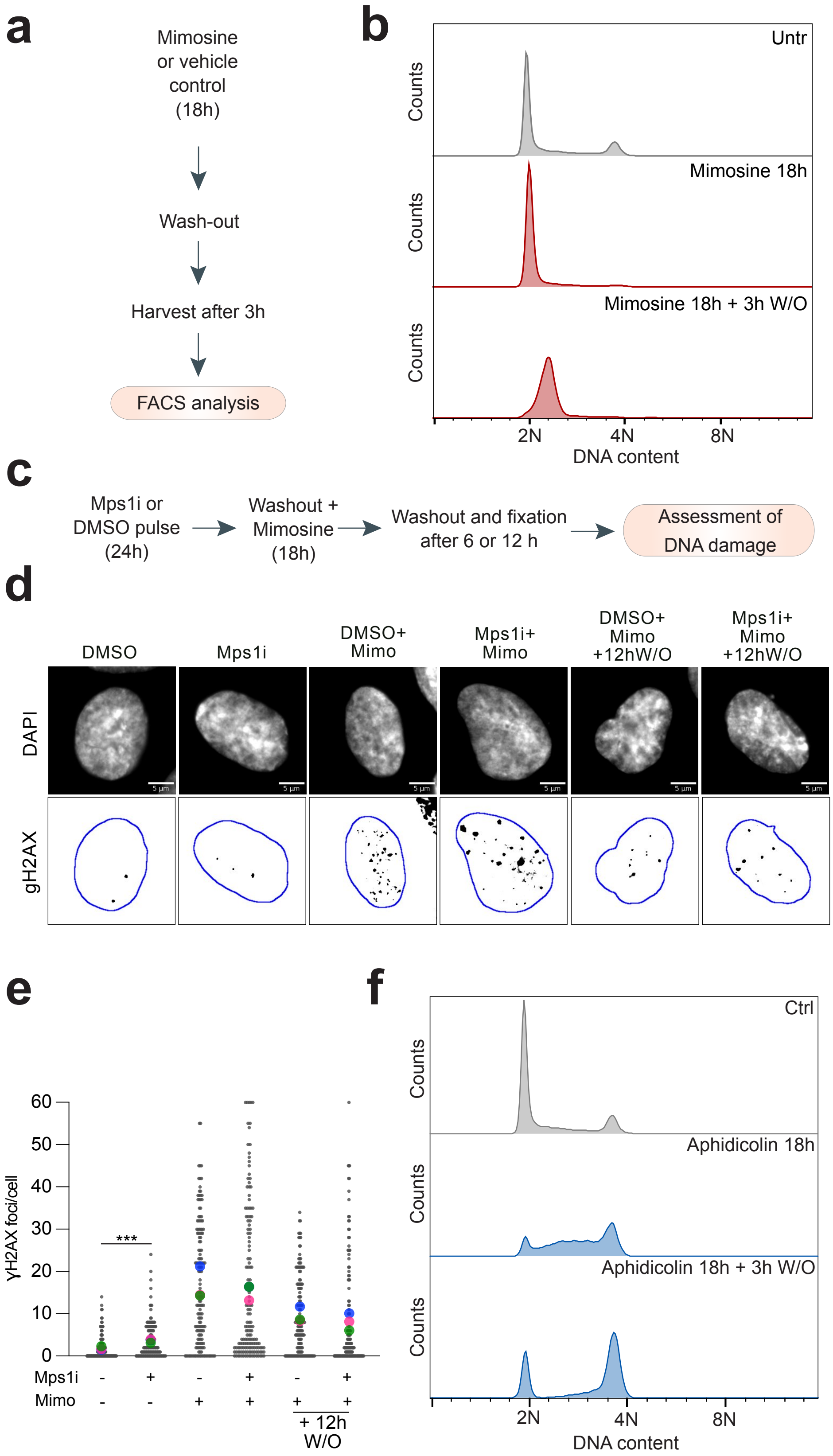


Supplementary Figure 2

**Supplementary Fig. 2: Impact of mimosine treatment and Mps1 inhibition on cell cycle and DNA damage. a**, Experimental setup for the FACS analysis of mimosine treated cells. **b**, Representative flow cytometry profiles of propidium-iodide stained cells. **c**, Experimental setup for the analysis of DNA damage upon mimosine treatment. **d,e**, Representative images (d) and relative quantification (e) of γH2AX foci per cell (n=150 for each sample). *** indicates p=0,0005. **f**, Representative flow cytometry profiles of propidium-iodide stained cells treated with aphidicolin as in panel a. Untr, untreated. Mps1i, Mps1i inhibition. Mimo, mimosine. W/O, washout. Scale bars, 5µm. LUT was inverted for γH2AX images. Blue borders in images are based on DAPI staining and define nuclei. Data are means of three biological replicates. Two-tailed unpaired Student's t-test was performed for data in panel e, where average values for each biological replicate are shown by colored dots (each color corresponds to a different biological replicate).

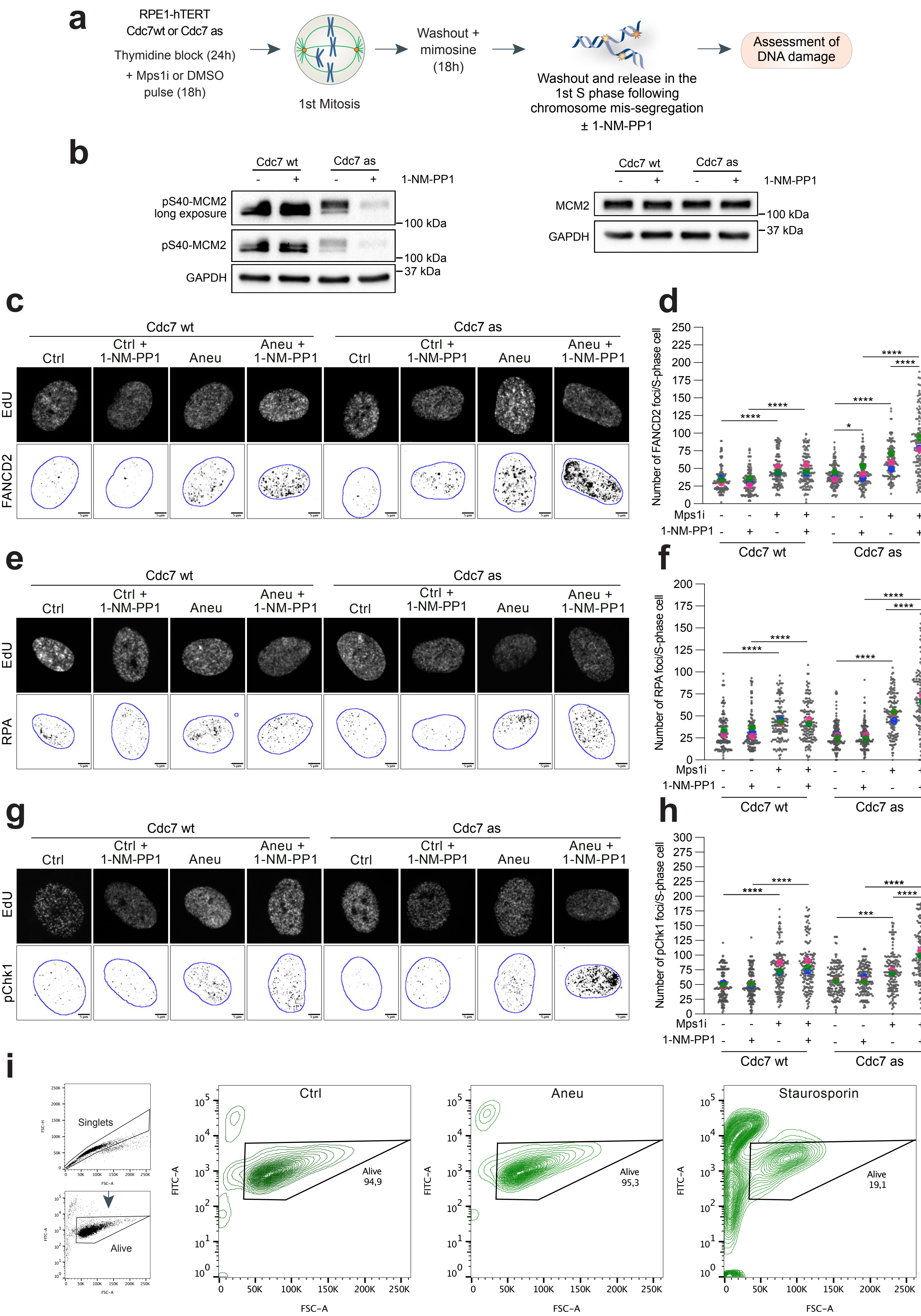


Supplementary Figure 3

**Supplementary Fig. 3: DDK inhibition leads to exacerbation of replication stress particularly in aneuploid cells. a,** Experimental workflow for the analysis of DNA replication stress markers in S-phase upon treatment with PP1 analog II, 1-NM-PP1, in hTERT RPE-1 cells expressing wild-type or analog-sensitive Cdc7. A short EdU pulse was performed before cell harvest in order to label S-phase cells. **b,** Western blot analysis of MCM2 and phoshoS40-MCM2 in cells expressing wt or as Cdc7. GAPDH was used as loading control. **c,d,** Representative images (c) and quantification (d) of FANCD2 foci per S-phase cell in control or aneuploid cells ± 1-NM-PP1 inhibitor in the two cell lines (n=153 for each sample). * indicates p=0,0373 and **** indicates p<0,0001. **e,f,** Representative images (e) and quantification (f) of RPA foci per S-phase cell in control or aneuploid cells ± 1-NM-PP1 inhibitor in the two cell lines (n=153 for each sample). **** indicates p<0,0001. **g,h,** Representative images (g) and quantification (h) of pChk1 foci per S-phase cell in control or aneuploid cells ± 1-NM-PP1 inhibitor in the two cell lines (n=156 for each sample). *** indicates p=0,0002 and **** indicates p<0,0001. **i,** FACS profiles showing the percentage of live cells in control and aneuploid cells. Gating strategy is illustrated on the left. Cells treated with staurosporin for 24 hours were used as a positive control of cell death. Ctrl, control (DMSO pulsed). Aneu, aneuploid cells (Mps1 inhibitor pulsed). wt, wild-type. as, analog-sensitive. Scale bars, 5µm. LUT was inverted for FANCD2, RPA and pChk1 images. Blue borders in images are based on DAPI staining and define nuclei. Data are means of three biological replicates. Two-tailed unpaired Student's t-test was performed for data in panel e, where average values for each biological replicate are shown by colored dots (each color corresponds to a different biological replicate). Source Data are provided as a Source Data file. Drawings of schemes were made by partially utilizing extracts of figures published elsewhere[1].

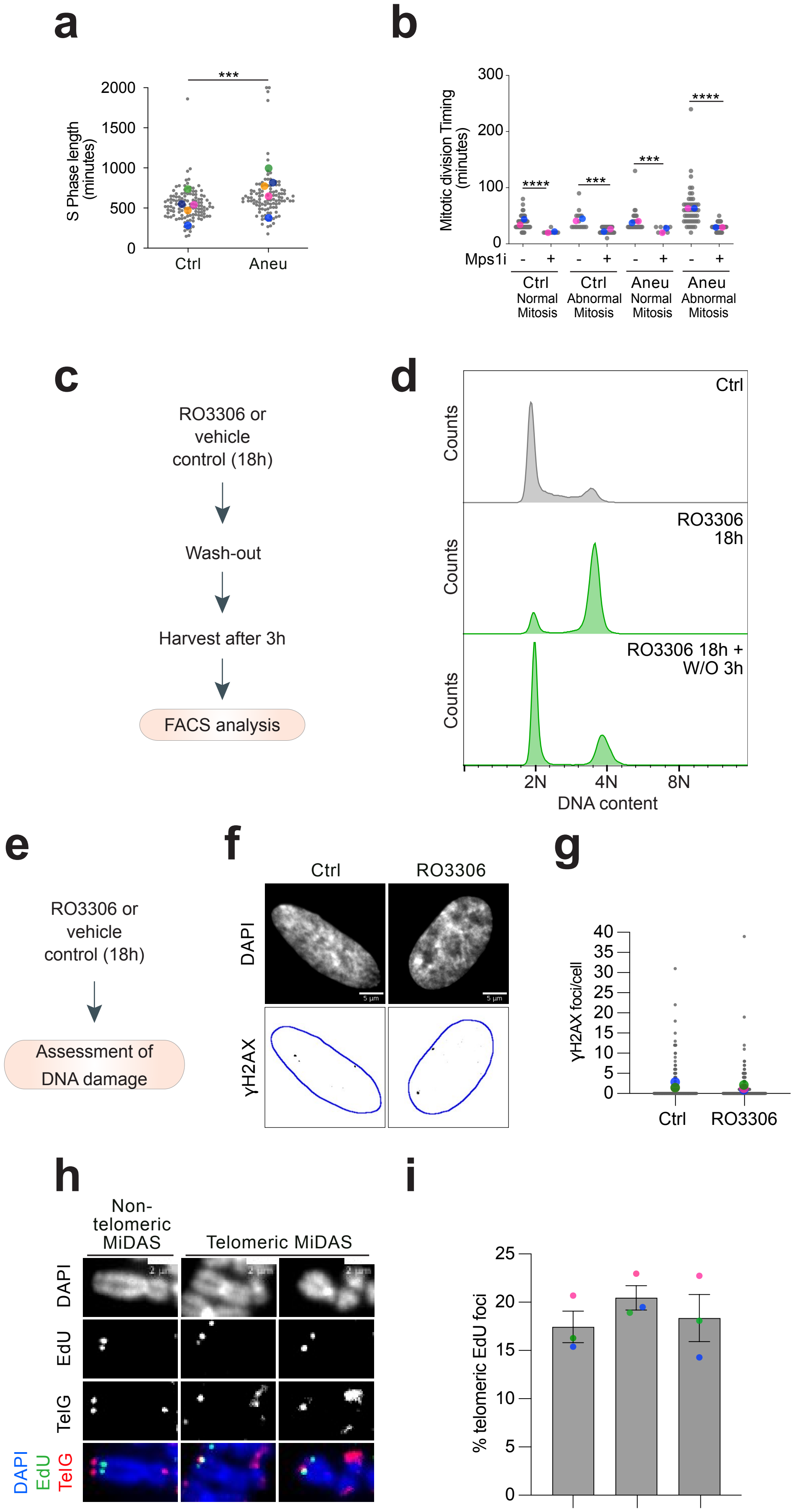


Supplementary Figure 4

**Supplementary Fig. 4: Effect of RO3306 treatment on cell cycle and DNA damage. a**, Quantification of S-phase duration by live-cell imaging in control (n=125) and aneuploid (n=106) cells. *** indicates p=0,0006. The graph refers to the experiment in Figure 3, panels a-d. **b**, Quantification of mitotic division timing upon SAC inhibition in control (n=54 for Ctrl Normal M, n=12 for Ctrl Normal M + Mps1i, n=16 for Ctrl Abnormal M, n=48 for Ctrl Abnormal M + Mps1i) and aneuploid (n=35 for Aneu Normal M, n=7 for Aneu Normal M + Mps1i, n=47 for Aneu Abnormal M, n=53 for Aneu Abnormal M + Mps1i) cells that underwent normal or abnormal mitosis. *** indicates p=0,0009 (Ctrl Abnormal M vs Ctrl Abnormal M + Mps1i) or p=0,0006 (Aneu Normal M vs Aneu Normal M + Mps1i) and **** indicates p<0,0001. **c**, Experimental setup for the FACS analysis of RO3306 treated cells. **d**, Representative flow cytometry profiles of propidium-iodide stained cells. **e**, Experimental setup for the analysis of DNA damage upon RO3306 treatment. **f,g**, Representative images (f) and relative quantification (g) of γH2AX foci per cell (n=150 for each sample). **h,i**, Representative images (h) and relative quantification (i) of telomeric EdU foci in control (n=111) and aneuploid (n=180) cells. Aphidicolin treated cells were used as positive control of replication stress (n=367). For illustration purposes, images were processed using Fiji. Briefly, images were filtered with median filter and background was subtracted using the dedicated Fiji function. Ctrl, control (DMSO pulsed). Aneu, aneuploid cells (Mps1 inhibitor pulsed). Mps1i, Mps1 inhibitor. W/O, washout. Scale bars, 5µm. LUT was inverted for γH2AX images. Blue borders in images are based on DAPI staining and define nuclei. Data are means of at least three biological replicates, except for data in panel b (two replicates). Error bars in panel i represent SEMs. Two-tailed unpaired Student's t-test was performed for data in panels a, b and g. Two-sided Chi square test was performed for data in panel i. In graphs, average values for each biological replicate are shown by colored dots (each color corresponds to a different biological replicate). Source Data are provided as a Source Data file. Drawings of schemes were made by partially utilizing extracts of figures published elsewhere[1].

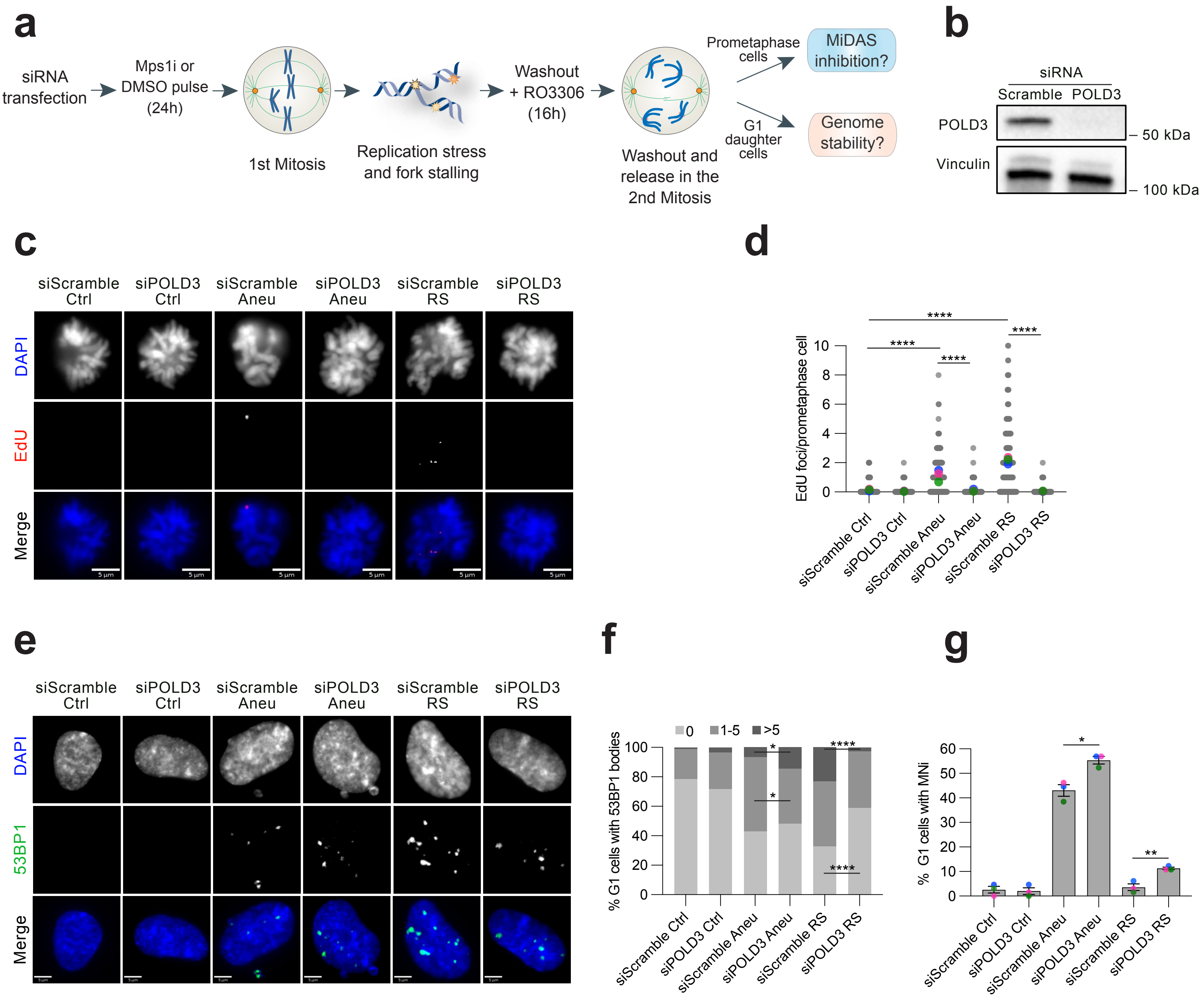


Supplementary Figure 5

**Supplementary Fig. 5: POLD3 depletion and consequent MiDAS inhibition result in a further increase in aneuploid cell genome instability. a,** Experimental workflow for the assessment of MiDAS occurrence in prometaphase cells and genome instability in the following G1 phase upon siRNA-mediated POLD3 depletion in hTERT RPE-1 cells. **b,** Western blot analysis of POLD3 in cells transfected with siRNA scramble or against POLD3. Vinculin was used as loading control. **c,d**, Representative images (c) and quantification (d) of EdU foci in prometaphase cells in control and aneuploid cells (n=150 for each sample). Cells treated with aphidicolin (RS, replication stress) were used as a positive control (n=150). **** indicates p<0,0001. **e-g,** Representative images (e) and quantification of 53BP1 body (f) and micronucleus (g) accumulation in control and aneuploid G1 cells upon POLD3 depletion (n=195 for each sample). Cells treated with aphidicolin (RS, replication stress) were used as a positive control (n=195). In panel f, * indicates p=0,0107 (siScramble Aneu vs siPOLD3 Aneu with 1-5 foci) or p=0,0133 (siScramble Aneu vs siPOLD3 Aneu with >5 foci) and **** indicates p<0,0001. In panel g, * indicates p=0,0197 and ** indicates p=0,0038. Ctrl, control (DMSO pulsed). Aneu, aneuploid cells (Mps1 inhibitor pulsed). RS, replication stress (aphidicolin treated cells). siScramble, transfected with non-targeting siRNA. siPOLD3, transfected with siRNA against POLD3. Scale bars, 5 μm. Data are means of three biological replicates. Error bars represent SEMs. Two-tailed unpaired Student's t-test was performed for data in panel d. Two-sided Chi square or Fisher's test was performed for data in panels f and g. In graphs, average values for each biological replicate are shown by colored dots (each color corresponds to a different biological replicate). Source Data are provided as a Source Data file.

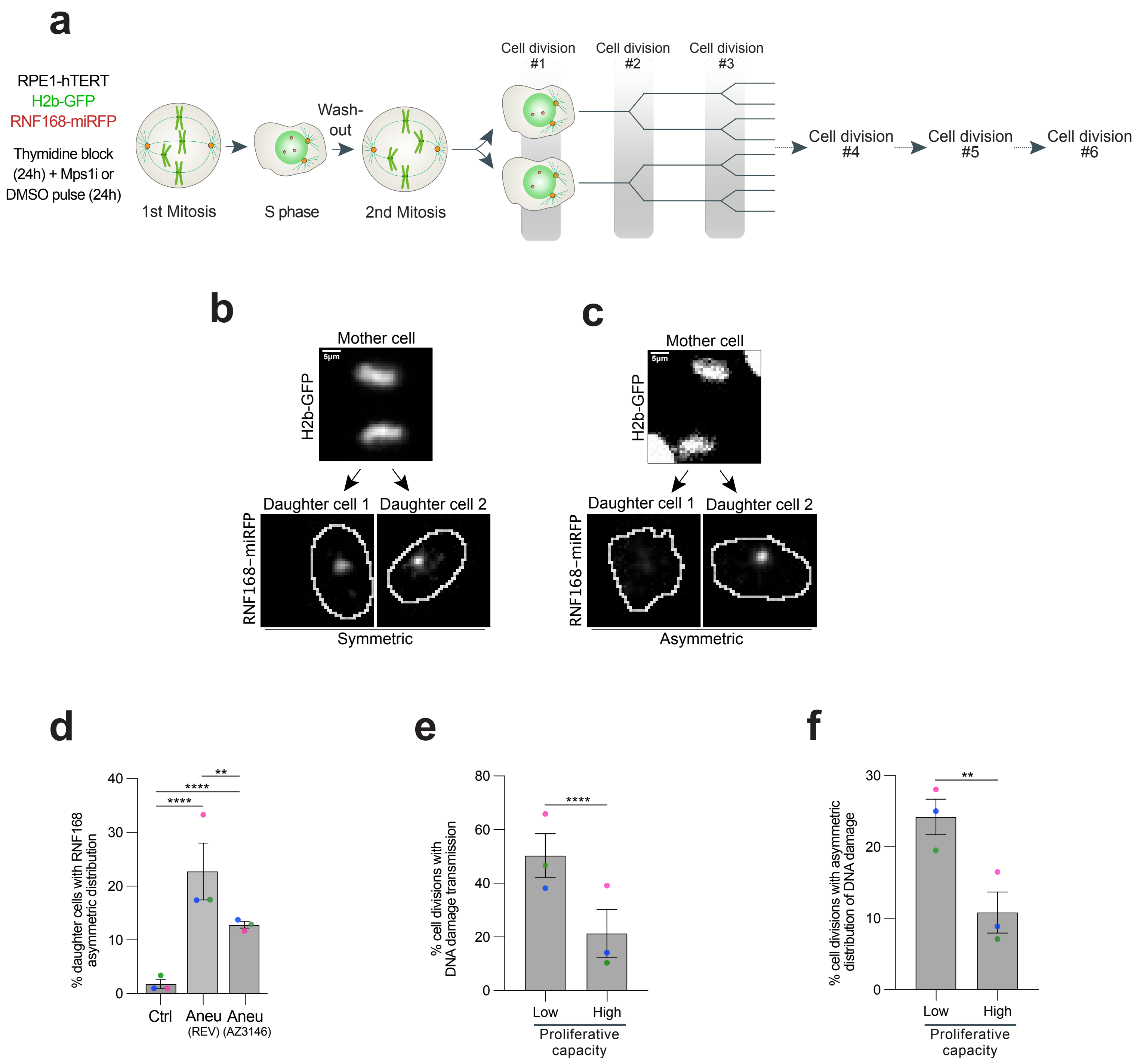


Supplementary Figure 6

**Supplementary Fig. 6: RNF168 asymmetric partitioning between daughter cells occurs at higher frequency in cells with reduced proliferative capacity. a,** Experimental workflow for the analysis of RNF168 distribution during cell division by live-cell imaging using hTERT RPE-1 cells expressing PCNA-chromobodies, RNF168-miRFP and H2b-GFP. **b,c**, Representative images of symmetric (b) and asymmetric (c) distribution of RNF168 between daughter cells. For presentation purposes, images were processed using Fiji. Briefly, images were filtered with gaussian blur, background was subtracted using the dedicated Fiji function and gamma transformation was applied to enhance the RNF168-miRFP signal. **d**, Quantification of RNF168 non-random distribution between daughter G1 cells in control (n=288) and aneuploid (n=459 for REV, n=250 for AZ3146) cells. ** indicates p=0,0027 and **** indicates p<0,0001. **e**, Quantification of cell divisions with DNA damage transmission in aneuploid cells (treated with reversine) that had divided less than 4 times ('Low', n=103) or at least 4 times ('High', n=286) over the almost 120 hour-time lapse experiment. **** indicates p<0,0001. **f**, Quantification of cell divisions with asymmetric distribution of DNA damage in aneuploid cells (treated with reversine) that had divided less than 4 times ('Low', n=103) or at least 4 times ('High', n=286) over the almost 120 hour-time lapse experiment. ** indicates p=0,0057. Ctrl, control (DMSO pulsed). Aneu, aneuploid cells (Mps1 inhibitor pulsed). Scale bars, 5µm. White borders in images are based on H2b-GFP signal and define nuclei. Data are means of three biological replicates. Error bars represent SEMs. Two-sided Chi square or Fisher's test was performed for data in panel d. Two-tailed unpaired Student's t-test was performed for data in panels e and f. In graphs, average values for each biological replicate are shown by colored dots (each color corresponds to a different biological replicate). Source Data are provided as a Source Data file.

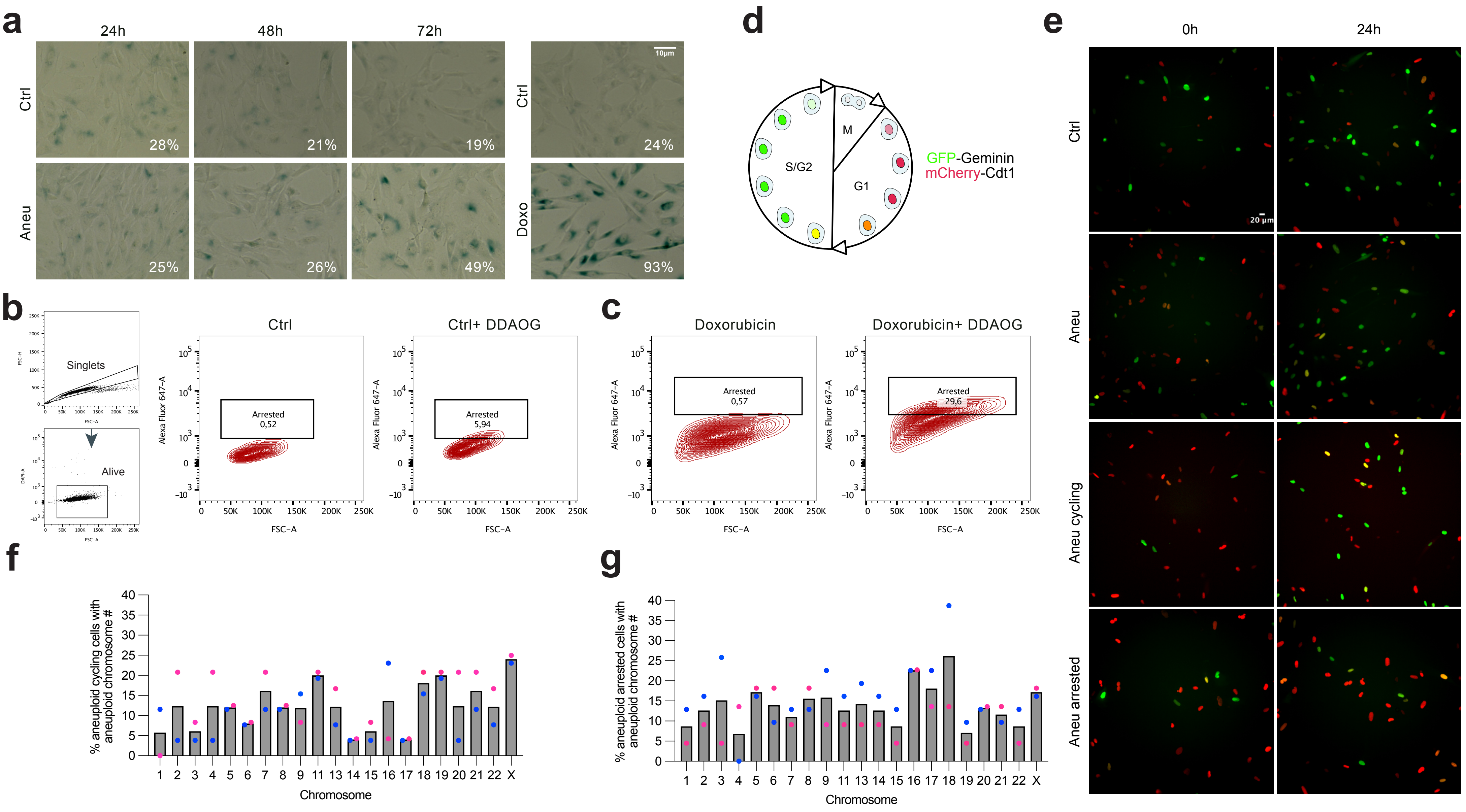


Supplementary Figure 7

**Supplementary Fig. 7: Separation and characterization of aneuploid cycling and arrested cells.** **a**, Senescence-associated β-galactosidase staining in control and aneuploid cells at different time points after the Mps1 inhibitor pulse (n=127 for Ctrl 24h, n=102 for Ctrl 48h, n=117 for Ctrl 72h, n=160 for Aneu 24h, n=160 for Aneu 48h, n=116 for Aneu 72h). Doxorubicin-treated cells were used as a positive control (n=250 for Ctrl, n=128 for Doxo). **b,c,** FACS profiles showing the percentage of DDAOG positive cells in DMSO (b) and doxorubicin (c) treated cells, used as a positive control. Gating strategy is illustrated on the left of panel c. **d,** Illustration depicting the change in nuclear color in the FUCCI cells. **e**, Representative images showing the starting (0h) and end point (24h) of the time-lapse in the four different samples (n=100 for each sample). **f,g** Histogram showing the percentage of cycling (n=50) or arrested (n=53) aneuploid cells with a given gained or lost chromosome. The graph refers to the experiment in Figure 5 j,k. Ctrl, control (DMSO pulsed). Aneu, aneuploid cells (Mps1 inhibitor pulsed). Aneu cycling, aneuploid cycling cells. Aneu arrested, aneuploid arrested cells. Doxo, doxorubicin. Scale bars, 10 or 20 µm. In graphs, average values for each biological replicate are shown by colored dots (each color corresponds to a different biological replicate). Source Data are provided as a Source Data file.

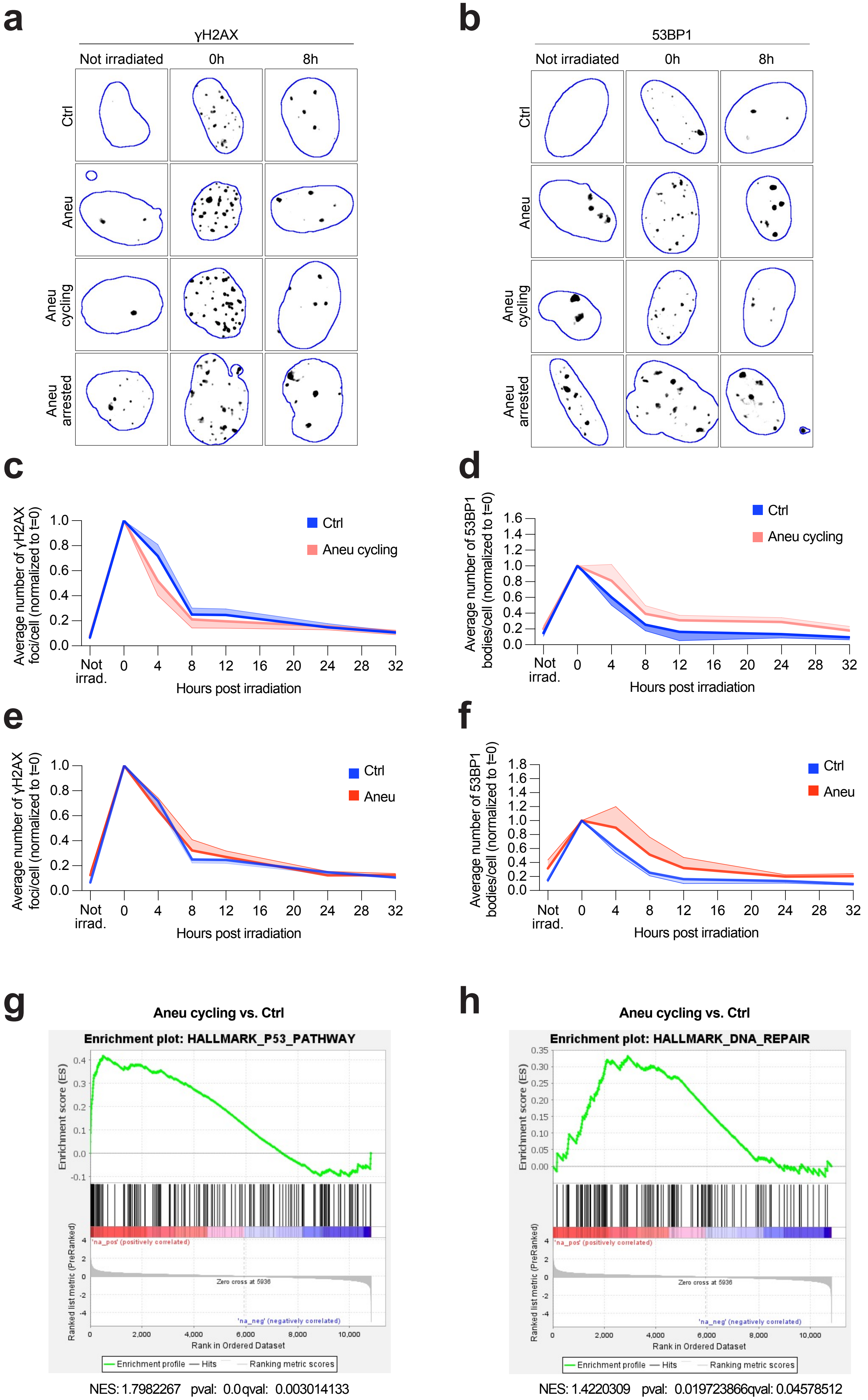


Supplementary Figure 8

**Supplementary Fig. 8: Kinetics of DNA damage repair in aneuploid cells and respective controls upon IR exposure. a-f** Representative images (a,b) and quantification of γH2AX (c,e) and 53BP1 bodies (d,f) per cell (n=150 for each sample in all data sets). Only EdU negative cells were analyzed in order to exclude the contribution of S-phase cells present in the non-arrested cell samples. **g,h**, Gene set enrichment analysis (GSEA) plots showing enrichment of p53 and DNA damage repair pathways in aneuploid cycling vs control cells. Ctrl, control (DMSO pulsed). Aneu, aneuploid cells (Mps1 inhibitor pulsed). Aneu cycling, aneuploid cycling cells. Aneu arrested, aneuploid arrested cells. Not irrad., not irradiated. LUT was inverted for γH2AX and 53BP1 images. Blue borders in images are based on DAPI staining and define nuclei. Data are means of three biological replicates. Shaded error bands in panels c-f represent SEMs. Source Data are provided as a Source Data file.

| gene_name | ensembl_gene_id | baseMean | log2FoldChange | lfcSE | stat | pvalue | padj | signature |
|---|---|---|---|---|---|---|---|---|
| UHRF1 | ENSG00000276043 | 992,6407794 | -1,463494234 | 0,23690646 | -6,177519319 | 6,51166E-10 | 1,46332E-07 | GOBP_DNA_REPAIR [569] |
| FOXM1 | ENSG00000111206 | 1019,397201 | -1,365225147 | 0,581676165 | -2,347053616 | 0,018922532 | 0,137644121 | GOBP_DNA_REPAIR [569] |
| PRIM1 | ENSG00000198056 | 474,8477874 | -1,364119404 | 0,245844729 | -5,548703086 | 2,87796E-08 | 3,52553E-06 | HALLMARK_DNA_REPAIR [150] |
| FANCE | ENSG00000112039 | 267,7712184 | -1,343504755 | 0,283509612 | -4,738833176 | 2,14952E-06 | 0,000111839 | REACTOME_DNA_REPAIR [332] |
| H2BC14 | ENSG00000273703 | 174,2119743 | -1,330320082 | 0,388364543 | -3,425441649 | 0,000613801 | 0,010177337 | REACTOME_DNA_REPAIR [332] |
| PTTG1 | ENSG00000164611 | 2092,055002 | -1,311532721 | 0,195952716 | -6,693108159 | 2,1848E-11 | 9,23032E-09 | GOBP_DNA_REPAIR [569] |
| CCNA2 | ENSG00000145386 | 2017,460861 | -1,280223222 | 0,584722995 | -2,189452498 | 0,028563967 | 0,180154541 | REACTOME_DNA_REPAIR [332] |
| RAD54L | ENSG00000085999 | 95,30515172 | -1,264984213 | 0,41289 | -3,063731777 | 0,002185948 | 0,028823949 | KEGG_HOMOLOGOUS_RECOMBINATION [28] |
| RMI2 | ENSG00000175643 | 198,7630434 | -1,256125997 | 0,317193169 | -3,96012941 | 7,49092E-05 | 0,002013207 | REACTOME_DNA_REPAIR [332] |
| EME1 | ENSG00000154920 | 125,8010331 | -1,251873356 | 0,378354632 | -3,308730094 | 0,000937201 | 0,014493003 | KEGG_HOMOLOGOUS_RECOMBINATION [28] |
| HMGB2 | ENSG00000164104 | 2568,528678 | -1,247776619 | 0,623843451 | -2,000143812 | 0,045484737 | 0,242631208 | GOBP_DNA_REPAIR [569] |
| H4C1 | ENSG00000278637 | 384,8643662 | -1,239926814 | 0,314814741 | -3,938591977 | 8,19612E-05 | 0,002148074 | REACTOME_DNA_REPAIR [332] |
| BLM | ENSG00000197299 | 392,269428 | -1,238186534 | 0,264789399 | -4,676118227 | 2,92356E-06 | 0,000142957 | KEGG_HOMOLOGOUS_RECOMBINATION [28] |
| PIF1 | ENSG00000140451 | 89,12425074 | -1,225055521 | 0,430923506 | -2,842860752 | 0,00447106 | 0,049708774 | GOBP_DNA_REPAIR [569] |
| H2AX | ENSG00000188486 | 1633,516624 | -1,214070301 | 0,226237375 | -5,366356033 | 8,03433E-08 | 7,50961E-06 | REACTOME_DNA_REPAIR [332] |
| MCM2 | ENSG00000073111 | 1471,107745 | -1,212075792 | 0,212945245 | -5,691959872 | 1,25589E-08 | 1,72269E-06 | GOBP_DNA_REPAIR [569] |
| PCLAF | ENSG00000166803 | 400,8196156 | -1,211648027 | 0,262104055 | -4,622774828 | 3,78641E-06 | 0,000178536 | REACTOME_DNA_REPAIR [332] |
| GINS2 | ENSG00000131153 | 692,4481938 | -1,198244536 | 0,216352556 | -5,538388641 | 3,05267E-08 | 3,62273E-06 | GOBP_DNA_REPAIR [569] |
| CHAF1B | ENSG00000159259 | 475,6941015 | -1,192518656 | 0,27551539 | -4,328319574 | 1,50251E-05 | 0,000547535 | GOBP_DNA_REPAIR [569] |
| MCM7 | ENSG00000166508 | 3970,843886 | -1,192254667 | 0,206152086 | -5,78337426 | 7,32169E-09 | 1,14107E-06 | GOBP_DNA_REPAIR [569] |
| MCM3 | ENSG00000112118 | 2961,430599 | -1,179418929 | 0,179290935 | -6,57824071 | 4,76047E-11 | 1,7338E-08 | GOBP_DNA_REPAIR [569] |
| POLE2 | ENSG00000100479 | 114,5677156 | -1,155770914 | 0,393054504 | -2,940485102 | 0,003276988 | 0,039064947 | KEGG_BASE_EXCISION_REPAIR [35] |
| H2BC7 | ENSG00000277224 | 244,6185528 | -1,153603703 | 0,356981821 | -3,231547476 | 0,001231219 | 0,018061294 | REACTOME_DNA_REPAIR [332] |
| FANCA | ENSG00000187741 | 276,1320378 | -1,147377048 | 0,323144533 | -3,550662107 | 0,000384263 | 0,007145405 | REACTOME_DNA_REPAIR [332] |
| ESCO2 | ENSG00000171320 | 604,1535975 | -1,131052097 | 0,254374819 | -4,446399614 | 8,73214E-06 | 0,000354727 | GOBP_DNA_REPAIR [569] |
| CHAF1A | ENSG00000167670 | 652,2068713 | -1,126431655 | 0,225719477 | -4,990405206 | 6,02528E-07 | 3,97744E-05 | GOBP_DNA_REPAIR [569] |
| MCM4 | ENSG00000104738 | 3502,112152 | -1,118147714 | 0,178359277 | -6,269075182 | 3,63199E-10 | 9,35635E-08 | GOBP_DNA_REPAIR [569] |
| HMGB1 | ENSG00000189403 | 4022,455871 | -1,097626305 | 0,161937686 | -6,778078246 | 1,21785E-11 | 5,59257E-09 | KEGG_BASE_EXCISION_REPAIR [35] |
| BRCA2 | ENSG00000139618 | 475,1108442 | -1,095058696 | 0,230034854 | -4,760403373 | 1,93206E-06 | 0,000103063 | KEGG_HOMOLOGOUS_RECOMBINATION [28] |
| DTL | ENSG00000143476 | 1403,11263 | -1,074765995 | 0,192323372 | -5,588327526 | 2,29267E-08 | 2,98953E-06 | REACTOME_DNA_REPAIR [332] |
| MCM5 | ENSG00000100297 | 1618,342543 | -1,072958858 | 0,202528225 | -5,297823822 | 1,17191E-07 | 1,01457E-05 | GOBP_DNA_REPAIR [569] |
| MCM6 | ENSG00000076003 | 1350,168882 | -1,070443972 | 0,191730505 | -5,583065521 | 2,36316E-08 | 3,04386E-06 | GOBP_DNA_REPAIR [569] |
| XRCC3 | ENSG00000126215 | 153,407276 | -1,067855756 | 0,409861183 | -2,605408369 | 0,009176482 | 0,083650383 | KEGG_HOMOLOGOUS_RECOMBINATION [28] |
| PAXX | ENSG00000148362 | 273,4354076 | -1,066297861 | 0,285542718 | -3,734284907 | 0,000188249 | 0,004057733 | GOBP_DNA_REPAIR [569] |
| ZWINT | ENSG00000122952 | 1208,569743 | -1,057468524 | 0,216065679 | -4,894199437 | 9,87067E-07 | 6,06128E-05 | HALLMARK_DNA_REPAIR [150] |
| H2BU1 | ENSG00000196890 | 164,4035717 | -1,0526433 | 0,425867637 | -2,471761665 | 0,013444912 | 0,107579665 | REACTOME_DNA_REPAIR [332] |
| DDX11 | ENSG00000013573 | 451,5202913 | -1,051661549 | 0,247909922 | -4,24211158 | 2,21427E-05 | 0,000754422 | GOBP_DNA_REPAIR [569] |
| H2AZ1 | ENSG00000164032 | 7719,34297 | -1,050492077 | 0,167503262 | -6,271472355 | 3,5765E-10 | 9,35635E-08 | REACTOME_DNA_REPAIR [332] |
| TRIP13 | ENSG00000071539 | 869,8179167 | -1,046154634 | 0,226576705 | -4,617220622 | 3,88914E-06 | 0,000182389 | GOBP_DNA_REPAIR [569] |
| RAD51 | ENSG00000051180 | 257,3393749 | -1,029007726 | 0,300546848 | -3,423784787 | 0,000617555 | 0,010223537 | HALLMARK_DNA_REPAIR [150] |
| DNA2 | ENSG00000138346 | 657,8128943 | -1,018641876 | 0,215024678 | -4,737325439 | 2,16557E-06 | 0,000112121 | REACTOME_DNA_REPAIR [332] |
| UBE2T | ENSG00000077152 | 667,2240543 | -1,00051634 | 0,248650561 | -4,023784771 | 5,72703E-05 | 0,001617349 | REACTOME_DNA_REPAIR [332] |
| CLSPN | ENSG00000092853 | 757,2423379 | -0,999979178 | 0,221806732 | -4,508335558 | 6,53382E-06 | 0,000289959 | REACTOME_DNA_REPAIR [332] |
| FANCD2 | ENSG00000144554 | 846,2549826 | -0,990211718 | 0,201856287 | -4,905528246 | 9,31762E-07 | 5,78898E-05 | REACTOME_DNA_REPAIR [332] |
| POLA1 | ENSG00000101868 | 675,0218176 | -0,984358017 | 0,20494672 | -4,802994741 | 1,5631E-06 | 8,6437E-05 | HALLMARK_DNA_REPAIR [150] |
| MCM8 | ENSG00000125885 | 296,7887492 | -0,977502892 | 0,267888176 | -3,648921385 | 0,000263344 | 0,005247992 | GOBP_DNA_REPAIR [569] |
| RMI1 | ENSG00000178966 | 149,590574 | -0,973469008 | 0,363808538 | -2,675772851 | 0,007455716 | 0,072645083 | REACTOME_DNA_REPAIR [332] |
| BRCA1 | ENSG00000012048 | 987,4511882 | -0,970000371 | 0,189075516 | -5,130227294 | 2,89393E-07 | 2,12261E-05 | REACTOME_DNA_REPAIR [332] |

Supplementary Table 1

**Supplementary Table 1: List of genes differentially expressed in arrested vs. cycling aneuploid cells.** Data were obtained by RNAseq (Fig. 6). Significance was determined with an empirical p-value, calculated using 1,000 permutations, using the default GSEA parameters. P-values were adjusted for multiple testing using the FDR method.
